# Effect of environmental stress factors on the uptake and survival of *Campylobacter jejuni* in *Acanthamoeba castellanii*

**DOI:** 10.1186/1471-2180-12-232

**Published:** 2012-10-11

**Authors:** Xuan Thanh Bui, Klaus Qvortrup, Anders Wolff, Dang Duong Bang, Carole Creuzenet

**Affiliations:** 1 National Veterinary Institute, Technical University of Denmark, Aarhus N, DK-8200, Denmark; 2 Department of Microbiology and Immunology, Infectious Diseases Research Group, University of Western Ontario, London, ON N6A 5C1, Canada; 3 Department of Biomedical Sciences, University of Copenhagen, Copenhagen N, 2200, Denmark; 4 BioLabChip group, Department of Micro and Nanotechnology, Technical University of Denmark, Kgs Lyngby, DK-2800, Denmark; 5 Laboratory of Applied Micro and Nanotechnology, National Food Institute, Technical University of Denmark, Søborg, DK-2860, Denmark

**Keywords:** *Campylobacter jejuni*, *Acanthamoeba castellanii*, Environmental stresses, Virulence

## Abstract

**Background:**

*Campylobacter jejuni* is a major cause of bacterial food-borne illness in Europe and North America. The mechanisms allowing survival in the environment and transmission to new hosts are not well understood. Environmental free-living protozoa may facilitate both processes. Pre-exposure to heat, starvation, oxidative or osmotic stresses encountered in the environment may affect the subsequent interaction of *C. jejuni* with free-living protozoa. To test this hypothesis, we examined the impact of environmental stress on expression of virulence-associated genes (*ciaB, dnaJ,* and *htrA*) of *C. jejuni* and on its uptake by and intracellular survival within *Acanthamoeba castellanii*.

**Results:**

Heat, starvation and osmotic stress reduced the survival of *C. jejuni* significantly, whereas oxidative stress had no effect. Quantitative RT-PCR experiments showed that the transcription of virulence genes was slightly up-regulated under heat and oxidative stresses but down-regulated under starvation and osmotic stresses, the *htrA* gene showing the largest down-regulation in response to osmotic stress. Pre-exposure of bacteria to low nutrient or osmotic stress reduced bacterial uptake by amoeba, but no effect of heat or oxidative stress was observed. Finally, *C. jejuni* rapidly lost viability within amoeba cells and pre-exposure to oxidative stress had no significant effect on intracellular survival. However, the numbers of intracellular bacteria recovered 5 h post-gentamicin treatment were lower with starved, heat treated or osmotically stressed bacteria than with control bacteria. Also, while ~1.5 × 10^3^ colony forming unit/ml internalized bacteria could typically be recovered 24 h post-gentamicin treatment with control bacteria, no starved, heat treated or osmotically stressed bacteria could be recovered at this time point. Overall, pre-exposure of *C. jejuni* to environmental stresses did not promote intracellular survival in *A. castellanii*.

**Conclusions:**

Together, these findings suggest that the stress response in *C. jejuni* and its interaction with *A. castellanii* are complex and multifactorial, but that pre-exposure to various stresses does not prime *C. jejuni* for survival within *A. castellanii*.

## Background

*Campylobacter jejuni* is a Gram-negative and microaerophilic bacterium that is considered the leading cause of human gastroenteritis worldwide [1,2]. *C. jejuni* colonises the intestine of most mammals and exists as a commensal in the gastrointestinal tract of poultry [3,4]. *C. jejuni* is typically transmitted to humans via consumption of undercooked food, unpasteurized milk, or contaminated water, or via contact with infected animals [2,5]. As it passes from host (commonly avian species) to human, *C. jejuni* must survive a great range of environmental stresses, including limited carbon sources, suboptimal growth temperatures, and exposure to atmospheric oxygen. Specifically, as a microaerophilic pathogen, *C. jejuni* must adapt to oxidative stress during transmission and colonization. In addition, this bacterium may struggle to accumulate adequate amounts of nutrients during residence in natural environments and during host colonization [4,6,7]. In food processing, *C. jejuni* must overcome high osmolarity conditions used for the inhibition of microbial growth in foods [8]. Furthermore, *C. jejuni* is able to adapt to a wide range of changing temperatures, from 42°C in avian hosts to ambient environmental temperatures or refrigeration conditions during food storage, higher temperatures during food processing and ultimately 37°C in the human host.

In order to survive these oxidative, starvation, osmotic and heat stresses, *C. jejuni* must be able to sense these changes and respond accordingly [9]. The ability of bacteria to alter protein synthesis is essential to respond and adapt to rapidly changing environments [10]. For example, several studies have focused on determining the mechanisms of *C. jejuni* survival at high temperatures. It has been shown that at least 24 proteins were up-regulated when cells were heat-shocked at temperatures ranging from 43 to 48°C [11], and a transient up- or down-regulation of 20% of *C. jejuni* genes was observed within 50 min of a temperature upshift from 37 to 42°C [12]. However, the genetic response of this bacterium to osmotic stress is not well known. Overall, despite the prevalence of *C. jejuni* infections, the molecular mechanisms that this pathogen uses to cause human disease, as well as the mechanisms utilized to adapt to environmental stresses encountered during both *in vivo* colonization and *ex vivo* transmission, are not well understood. A better understanding of these mechanisms is required in order to facilitate the development of appropriate intervention strategies to reduce the burden of *C. jejuni*-associated diseases [13].

Aquatic environments are reservoirs for *C. jejuni*[7,14,15] and contaminated drinking water has been implicated in several *C. jejuni* outbreaks [16-18]. *Acanthamoeba* spp. are free-living amoebae which can be found widely in water [19-21]. They have evolved efficient mechanisms to phagocytose and kill bacteria that they use as a source of nutrients [22,23]. However, the relationship of amoeba with bacteria can be complex. We and others have indicated that amoebae can promote the survival of *C. jejuni*[24-28] and our study specifically showed that the bulk of this growth was extracellular. We also showed that while the majority of internalized *C. jejuni* does not survive ingestion by *A. castellanii* beyond 5 h, a very small number of bacterial cells are able to survive intracellularly and are thereby protected from external disinfectant killing during this time frame [27]. During this period, chicks may still get contaminated by *Campylobacter* from infected amoebae present in the water source, as it has been reported that intra-amoeba *Campylobacter* can colonize broiler chickens and may represent a significant environmental source of transmission [29].

Although the mechanisms of survival of *C. jejuni* outside the host are not fully understood, it has been proposed that stress-adapted *C. jejuni* can survive environmental stresses better than non-stressed cells [10,30]. Likewise, pre-exposure to stress may affect the interaction of stressed *C. jejuni* cells with amoeba. To date, little is known about the interaction of stressed *C. jejuni* and *A. castellanii*, but this needs to be investigated as both of these organisms occupy a similar ecological habitat [21,31,32]. The importance of the interplay between *C. jejuni* and amoeba under stress conditions was recently highlighted by the fact that co-incubation with amoeba increases acid tolerance and survival of *C. jejuni*[24,26,27,33]. Therefore, the interactions between *C. jejuni* and *Acanthamoeba* are relevant to the transmission of *C. jejuni* from the environment to new hosts.

Several genes and the encoded proteins have been shown to be important for *C. jejuni* to adapt to environmental changes and to facilitate its interactions with eukaryotic cells. Examples of potential relevance to this study are the CiaB protein, which enhances invasion of eukaryotic cells [34,35], and the HtrA protein that degrades and prevents aggregation of periplasmic proteins that misfold during stress [36,37]. Another example is DnaJ, which aids in protein folding and plays a role in *C. jejuni* thermotolerance and in chicken colonization [11,38]. Transcription of *dnaJ* is up-regulated upon temperature stress [12].

The aims of this study were: 1) to investigate the effect of environmental stress factors, namely osmotic, heat, oxidative and low nutrient stresses on the survival of *C. jejuni* and on the transcription of virulence-associated genes (*htrA*, *ciaB*, *dnaJ*) that are known to play important roles in the stress response of *C. jejuni*, its interactions with eukaryotic cells and the colonization of chickens [11,35,38,39]; and 2) to investigate the effect of these stresses on the uptake of *C. jejuni* by *A. castellanii* and on its intracellular survival. The underlying hypothesis was that pre-exposure to stress may prime *C. jejuni* for resistance to further environmental pressure such as phagocytosis by amoeba and intracellular killing, and this priming could be monitored via the levels of transcription of the chosen virulence-associated genes.

## Results

### Effect of environmental stresses on the survival of *C. jejuni*

**Figure 1:**
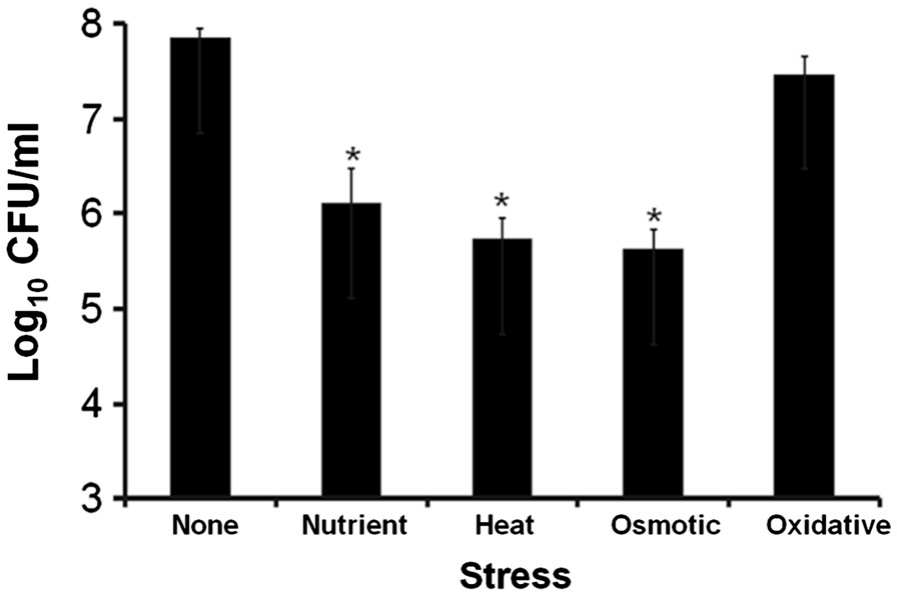
**Survival of *C. jejuni *cells exposed to environmental stresses in pure planktonic culture in the absence of any amoeba.** Survival was determined by counting colony forming units (CFU). Data are means and standard errors of three independent experiments. The treatment was statistically compared with the no stress control. (*), p < 0.05.

As shown in Figure 1, exposure to low nutrient, heat and osmotic stresses strongly decreased the survival of *C. jejuni* in pure planktonic cultures (no amoeba) as assessed by colony forming unit (CFU) counting. While in the conditions tested, 7.9 log_10_ CFU/ml were measured in the absence of stress, only 6.1, 5.7 and 5.6 log_10_ CFU/ml were measured after low nutrient, heat or osmotic stress, respectively, which amounted to ~ 60, 105 and 144 fold reductions in the CFU numbers. The results were statistically significant, with *p* values less than 0.05 as per *t-*test. Heat and osmotic stresses reduced the survival of *C. jejuni* the most. In contrast, exposure of *C. jejuni* to hydrogen peroxide (oxidative stress) for 15 min only triggered a 2 fold (not statistically significant) decrease of survival of *C. jejuni* since 7.4 log_10_ CFU/ml were recovered.

### Transcription of virulence genes in *C. jejuni* under environmental stresses

**Figure 2:**
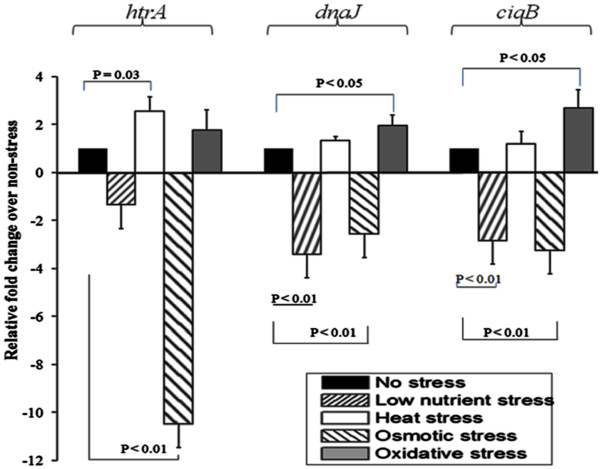
**qRT-PCR analysis of the impact of the various stresses on transcription of virulence-associated genes of *C. jejuni *.** Total RNA was isolated, and the expression of *ciaB*, *dnaJ* and *htrA* was measured immediately after exposure to each stress. All data were normalized to the level of expression of the 16S rRNA gene and are presented relatively to the non-stress control. Therefore, the non-stressed condition has a fold value of 1. Data are representative of three independent experiments from three RNA extracts.

Three virulence-related genes, *htrA*, *dnaJ* and *ciaB,* were chosen as reporters to monitor transcriptional regulation that occurred after exposure of *C. jejuni* to various stresses*.* First, quantitative real-time RT-PCR analyses were performed to check the basal level of transcription of each of the selected gene when the bacteria were grown *in vitro* in optimal conditions of osmolarity and nutrient availability (in Trypic soy agar with 5% sheep blood) and of temperature (37°C) and oxygen concentration (5%) [27]. All three genes were transcribed constitutively at high levels, with respective levels of transcription of *htrA*, *dnaJ*, and *ciaB* only 7.6, 12.5, and 7.5 fold lower than the very highly transcribed 16S rRNA internal control (data not shown). Secondly, the impact of stress on the levels of expression of these genes was tested. Control experiments indicated similar levels of transcription of the 16S rRNA gene under all stresses tested, apart from a small and non significant 1.7 fold increase in osmotic stress conditions (data not shown). Therefore, the 16S rRNA gene was again used as the reference to determine the change in transcription levels of virulence-associated genes induced by stress relative to bacterial cells in the absence of any stress. As shown in Figure 2, the transcription of *dnaJ* and *ciaB* was not affected by heat stress and only slightly altered after exposure to the other stresses. A modest up-regulation was observed under oxidative stress (~2.7 and 2 fold for *ciaB* and *dnaJ*, respectively, p < 0.05) while a modest down-regulation (~2.8 to 3.2 fold, *p <* 0.01) was observed for both genes under low nutrient or osmotic stresses. The transcription of *htrA* was moderately up-regulated under oxidative stress and slightly down-regulated under low nutrient stress, but the change was not statistically significant (*p >* 0.05). In contrast, transcription of *htrA* was up-regulated 2.5 fold under heat stress (*p* = 0.03) and down-regulated ~10 fold under osmotic stress (*p <* 0.01).

Overall, the qRT-PCR experiments showed that the transcription of the three virulence-associated genes chosen was only slightly up-regulated under heat and oxidative stresses, but tended to be down-regulated under low nutrient and osmotic stresses, with *htrA* showing the most down-regulation in response to osmotic stress.

### Effect of *htrA* on the uptake of *C. jejuni* by *A. castellanii* and its intracellular survival

**Figure 3:**
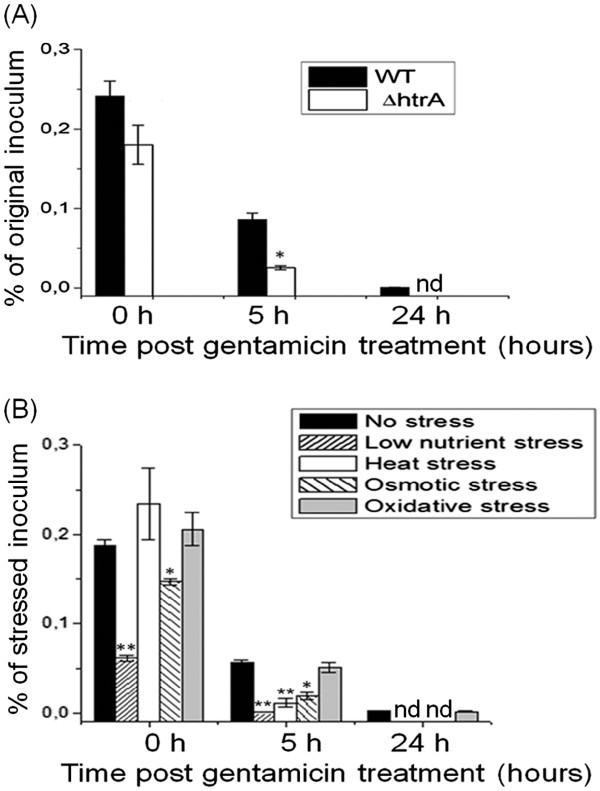
**Intracellular survival rates of *C. jejuni *cells within *A. castellanii *.** Intracellular survival rates were determined by colony forming unit (CFU) counting at 0, 5, and 24 h post gentamicin treatment at 25°C in aerobic conditions. Panel **A**: comparison of wild-type (WT) and *htrA* mutant. Panel **B**: comparison of stressed and non-stressed wild-type bacteria. Data are means and standard errors of three independent experiments. Statistically significant differences concern comparisons between control and treatment groups. (*) p < 0.05; (**) p < 0.01; nd, none detected.

We showed above that the transcription of at least one of the few virulence-associated genes tested (*htrA*) was affected by osmotic stress at a level that could be biologically significant (10 fold). Transcriptional regulation of virulence-associated genes upon pre-exposure to stress may affect interactions of *C. jejuni* with host cells, including phagocytosis and the ability of *C. jejuni* to survive in host cells after internalization. To determine whether this was the case for interactions with amoeba, we tested the biological importance of the stress-related gene for which we had observed the largest transcriptional variations (*htrA*) using the *htrA* mutant that was previously described [39]. Both bacterial uptake and intracellular survival were measured after interactions of 2 × 10^8^ bacteria with amoeba at a multiplicity of infection of 100 for 3 h at 25°C (see Methods section for more details). The intracellular bacteria were enumerated using the gentamicin protection assay that we previously optimized for amoebae [27]. Immediately after elimination of extracellular bacteria by gentamicin treatment (0 h post gentamicin treatment), no statistically significant difference was observed in the counts of internalized wild-type or *htrA* mutant bacteria (Figure 3A), with 0.24 and 0.18% of the original inoculum recovered, respectively. The counts of internalized bacteria recovered 5 h post gentamicin treatment decreased significantly to 0.08 and 0.025% of the original inoculum for the wild-type and the *htrA* mutant, respectively. This decrease in intracellular survival was significantly greater for the *htrA* mutant (~7 fold) compared to the wild-type strain (~3 fold) (Figure 3A). While no *htrA* mutants were detected at 24 h, ~1 × 10^3^ CFU/ml of wild-type bacteria were recovered at this time point, representing a ~300 fold reduction compared with the 0 h time point. These data indicate that *htrA* is important for intra-amoebae survival in the 24 h time frame studied, but not for the uptake step. This suggests that pre-exposure to stress, via its transcriptional regulation on virulence-associated genes, may affect survival of intra-amoeba bacteria.

### Uptake of stressed *C. jejuni* by *A. castellanii* and intracellular survival

To examine the impact of pre-exposure to stressful environments on the degree of phagocytosis by amoebae and on the intracellular survival of wild-type *C. jejuni* in amoebae, stressed and non-stressed *C. jejuni* cells were co-cultured with *A. castellanii*. Approximately 4.5 × 10^8^ CFU/ml bacteria were subjected to either the stress or control treatments before interactions with amoeba. The survival data presented in Figure 3B were normalized to account for the number of bacteria that had survived exposure to the stress tested (or to the control treatment) before inoculation of the amoeba. Immediately after elimination of extra-amoeba bacterial cells by gentamicin treatment, approximately 0.18% of the original non-stressed bacterial inoculum was recovered as internalized bacteria*,* but only ~0.06 and 0.14% of the *C. jejuni* inoculum pre-exposed to low nutrient and osmotic stresses were recovered, respectively (Figure 3B). No statistically significant differences were obtained with *C. jejuni* pre-exposed to heat and oxidative stresses compared with non-stressed bacteria. At 5 h post gentamicin treatment, only 0.06% of the original inoculum was obtained for non-stressed *C. jejuni*. Pre-exposure of bacteria to heat, starvation or osmotic stresses exacerbated the bacterial susceptibility to intracellular killing, since a significant decline of the number of surviving bacteria was observed upon pre-exposure to these stresses 5 h post-gentamicin treatment (Figure 3B). At 24 h post gentamicin treatment, a few internalized bacteria (~1.5 × 10^3^ CFU/ml) were observed with non-stressed inoculum. No bacteria that had been pre-exposed to heat, starvation or osmotic stress were detected. In contrast, pre-exposure to oxidative stress had no impact on internalization or intracellular survival of *C. jejuni* under the conditions and time frame studied.

### Effect of pre-exposure to stress on sub-cellular location of internalized bacteria

**Figure 4:**
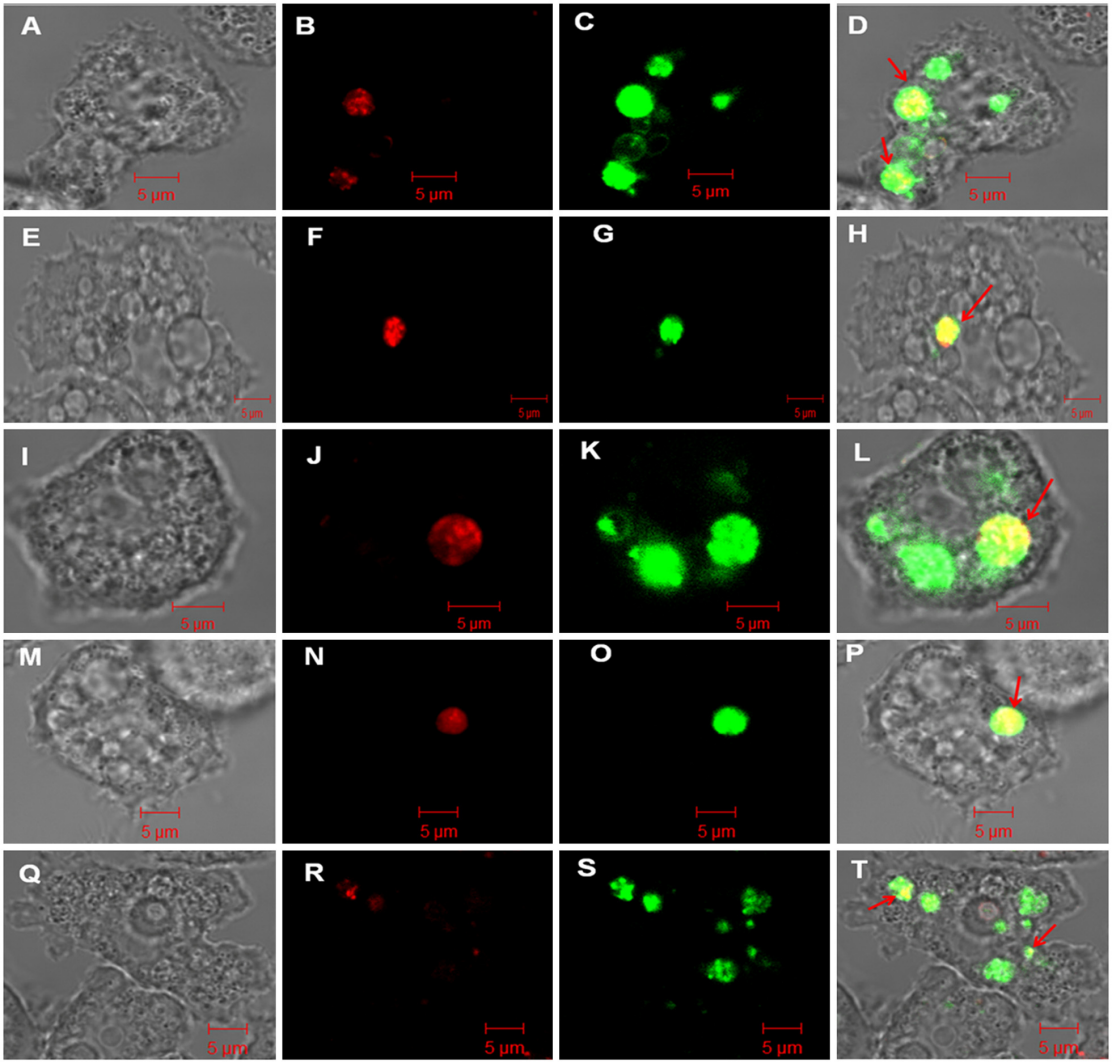
**Confocal microscopy analysis of stressed and non-stressed *C. jejuni *cells within acidic organelles of *A. castellanii *observed immediately after gentamicin treatment.** Control *C. jejuni* (**A-D**), *C. jejuni* pre-exposed to osmotic stress (**E-H**), heat stress (**I-L**), hydrogen peroxide (**M-P**), or starvation stress (**Q-T**). The multiplicity of infection was 100:1 (bacteria:amoeba). (**A, E, I, M**, Q) differential interference contrast image; (**B, F, J, N, R**) *C. jejuni* stained with CellTracker Red; (**C, G, K, O, S**) acidic amoeba organelles stained with LysoSensor Green; (**D, H, L, P, T**) corresponding overlay. Scale bar = 5 μm.

A detailed observation of *C. jejuni* cells internalized within the amoebae was carried out by confocal laser scanning microscopy (CLSM). In the absence of any stress, live *C. jejuni* cells were detected by CellTracker Red staining inside the trophozoites immediately after gentamicin treatment (Figure 4A, B). The intracellular bacteria were distributed as clusters within acidic vacuoles as observed by the simultaneous staining of acidic vacuoles by LysoSensor Green DND-189 (Figure 4C, D). Pre-exposure of bacteria to low-nutrient, heat, osmotic or oxidative stresses did not qualitatively alter the sub-cellular location of internalized bacteria, as all were also recovered in acidic vacuoles (Figure 4E to T).

**Figure 5:**
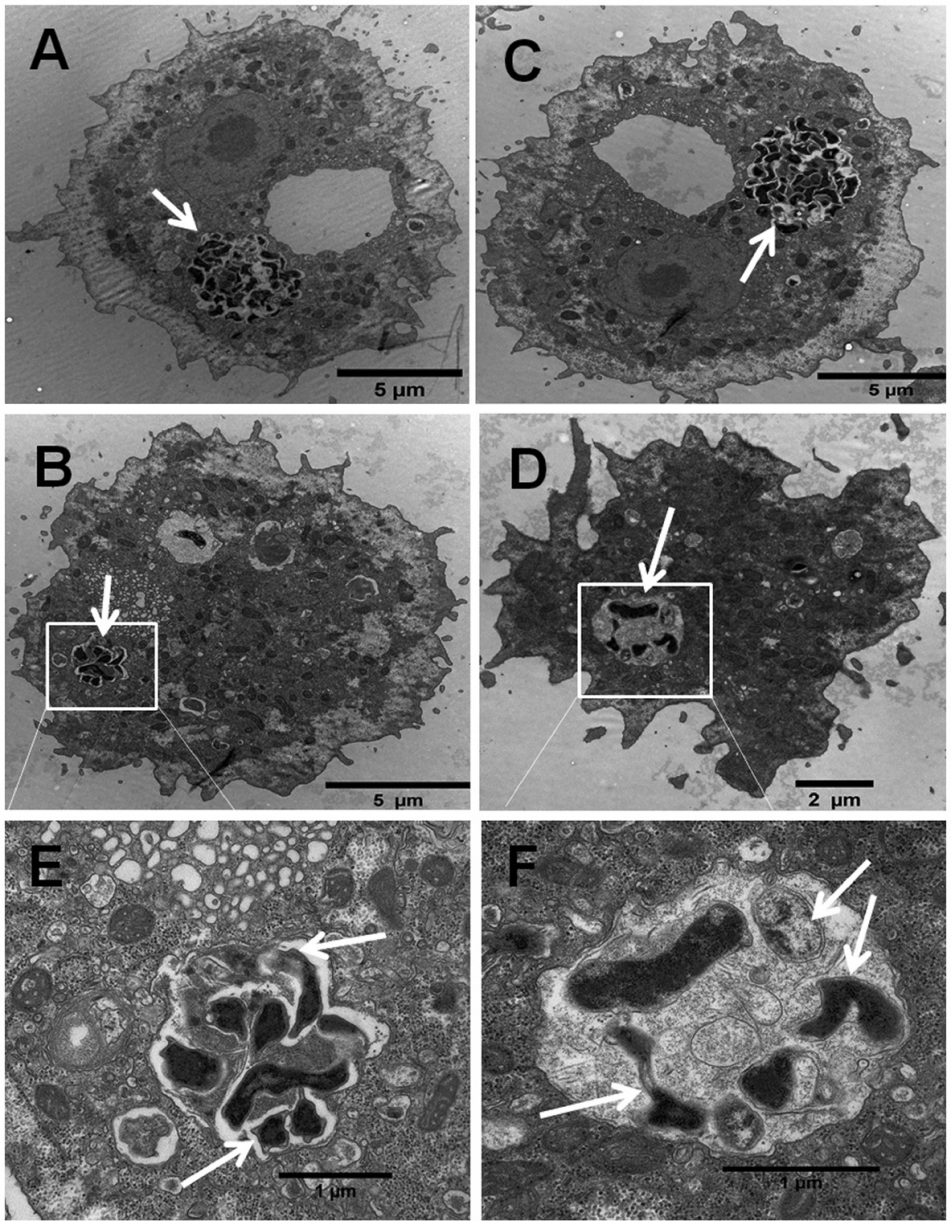
**TEM of control *C. jejuni *and *C. jejuni *pre-exposed to heat stress within vacuoles of *A. castellanii *trophozoites at different time points.** At 0 h after gentamicin treatment, control *C. jejuni* (**A**) and *C. jeuni* pre-exposed to heat stress (**C**). At 5 h after gentamicin treatment, control *C. jejuni* (**B** and with zoom out in **E**) and heat stressed *C. jejuni* (**D** and with zoom out in **F**). The white arrows show *C. jejuni* cells inside amoeba vacuoles.

In addition to the viable count assay for the quantification of intracellular bacteria and CLSM analyses reported above, TEM was also used to more precisely assess the effect of heat stress on intracellular location of *C. jejuni* within *A. castellanii*. Heat stress was selected for TEM studies because it decreased intracellular survival of *C. jejuni*, but it did not affect uptake. Therefore this heat stress allowed visualization of numerous internalized bacteria at early time points. As shown in Figure 5, sections of infected *A. castellanii* cells obtained right after gentamicin treatment showed that *C. jejuni* cells were confined to tight vacuoles within the amoebae, whether they had been heat-stressed or not prior to co-culture with amoebae (Figure 5A, C). At 5 h post gentamicin treatment, fewer internalized bacteria could be seen inside the amoeba vacuoles (white arrows Figure 5B, D, E, F), and heat stress reduced the number of bacteria present in the vacuoles (Figure 5D, F) compared with control bacteria (Figure 5B, E). This corroborated the survival and CLSM data described above.

## Discussion

### Effect of pre-exposure to stress on survival of *C. jejuni*

Although *C. jejuni* has strict growth requirements [40-42], it has developed mechanisms for survival in diverse environments, both inside and outside the host, where it is subjected to various stresses [40,43]. In agreement with prior studies [4,7,44-48], our data showed that heat, low nutrient and osmotic stresses significantly reduced the survival of *C. jejuni* in the absence of amoeba (Figure 1), as assessed by colony forming units counting. *C. jejuni* is known to turn into coccoid cells under sub-optimal culture conditions, which correlates with decreased culturability [6,49]. However, we observed by CLSM microscopy that, under the stress conditions applied, only a small proportion of the cell population turned into coccoid cells (Data not shown). Therefore, coccoid formation could not account for the described decrease in viability.

Pre-exposure to oxidative stress did not affect the survival of *C. jejuni* in comparison with non-stressed cells. This could reflect the fact that *C. jejuni* possesses mechanisms which can eliminate reactive oxygen species to prevent cellular damage [42,50]. While these systems are not as developed as in aerobic bacteria and only allow survival of *C. jejuni* under moderate oxidative stress, their existence could explain why the limited oxidative stress imposed had no effect on the survival of *C. jejuni*. The oxidative treatment applied in this study was nevertheless shown previously to be sufficient to induce considerable transcriptional regulation [13], which we also observed for the *ciaB* gene (see below).

### Effect of pre-exposure to stress on the transcription of *ciaB, htrA* and *dnaJ*

The transcription of virulence genes is modulated by different stresses in many bacterial pathogens [51-53]. As a microaerophilic bacterium, *C. jejuni* must adapt to oxidative stress during transmission and infection [7] and, consistent with this idea, our qRT-PCR data showed that oxidative stress increased the transcription of the *ciaB* gene (2.7 fold). This is reminiscent of a previous report that culture with bile acid deoxycholate primes *C. jejuni* to invade epithelial cells by stimulating the synthesis of Cia proteins [54]. Likewise, transcriptional regulation of *ciaB* was observed under low nutrient and osmotic stresses, but in contrast to oxidative stress, slight decreases (2.8 and 3.2 fold) of transcription were observed. This is in agreement with a prior report of decreased transcription of *ciaB* under starvation stress [10].

HtrA is important for stress tolerance and survival of Gram-negative bacteria as it degrades periplasmic proteins that misfold under stress [36,37]. HtrA is also important for the virulence of *C. jejuni*[39,55-57], and we showed herein that HtrA is important for intra-amoeba survival of *C. jejuni* by using the *htrA* mutant (Figure 3). However, limited data are available regarding *htrA* transcriptional regulation during environmental stress in *C. jejuni*. Our qRT-PCR results showed that heat, oxidative and low nutrient stresses only slightly altered *htrA* transcription. Because the basal level of transcription of *htrA* is rather high and only limited variations in transcription were observed under stress, the levels of HtrA protein may be sufficient to maintain a proper periplasmic environment under all conditions tested. Surprisingly, osmotic stress heavily repressed the transcription of *htrA* (~10 fold). Such down-regulation is counter-intuitive since hyper osmotic stress likely causes aggregation of proteins upon loss of cellular fluids by osmosis. Other stress-response mechanisms may be up-regulated to counter-act the down-regulation of transcription of *htrA.* Their identity is up for debate since *C. jejuni* does not have the traditional CpX and RseA/B stress response systems [39].

While the DnaJ *chaperone* plays a role in *C. jejuni* thermo-tolerance and in chicken colonization [11,38], and *dnaJ* transcription was shown previously to be enhanced under heat stress [12], we did not observe any effect of heat stress on the transcription of *dnaJ*. This discrepancy is likely due to the very different heat stresses applied. Our study was geared at studying changes occurring during the chain of transmission (change from ambient to chicken temperature of 42°C) and during food processing (warm up to 55°C) as also reported by Gundogdu et al. [13], while available transcriptional studies are more focused on changes occurring during chicken/human host transition (42–37°C variations) [12].

Altogether, although the levels of transcriptional regulation were generally low and varied between the three virulence-associated genes tested, similar trends were observed: up-regulations upon oxidative and heat stress versus down-regulation upon low nutrient and osmotic stresses. This indicates that stress-response mechanisms other than those encoded by the three genes investigated are more important in assisting cells to overcome low nutrient and osmotic stresses.

### Effect of pre-exposure to stress on uptake of *C. jejuni* by amoeba

Since the modulation of virulence genes in response to stresses is a common phenomenon of pathogenic bacteria, it is important to get insight into the influence of these conditions on the interaction of bacteria with other organisms, such as amoebae, which exist in similar habitats [21,31,32]. Beyond the data presented herein, no data are currently available to determine whether pre-exposure to environmental stresses might affect bacterial uptake or intracellular killing by amoeba. Other *C. jejuni*/amoeba studies were performed using bacteria grown in optimal culture conditions (temperature, media and atmospheric conditions) which are not adapted to stressful conditions [24-28], or simply probe the ability of *C. jejuni* to sustain stressful conditions during or after interactions with amoeba [33]. Stress-induced bacterial adaptation to enhance the bacteria’s ability to survive a subsequent interaction with amoeba, and amoeba-mediated enhanced bacterial resistance to stress are complementary mechanisms that are important for the survival of *C. jejuni* in the environment. Our data showed that low nutrient and osmotic stresses were the strongest factors which significantly affected the survival of *C. jejuni* (Figure 1, decreased survival in pure cultures without amoeba) and the transcription of three virulence-associated genes (Figure 2), and also reduced the uptake of the bacterium by *A. castellanii* (Figure 3). Our findings are consistent with previous studies that reported that starvation strongly affected *C. jejuni* invasion in Caco-2 and macrophages [6,58]*.*

In contrast, our data showed that heat and oxidative stresses did not affect the uptake of *C. jejuni* by amoebae. These findings differ from previous studies that reported that pre-exposure of *C. jejuni* to oxidative stress increased the invasion of *C. jejuni* in intestinal cells [45,47], and that heat stress reduced the invasion of *C. jejuni* in Caco-2 and macrophages. These discrepancies are likely due to cell line-specific mechanisms of uptake and killing, variations in the nature and abundance of appropriate eukaryotic receptors [59], and differences in the experimental set up used to apply the heat stress as indicated above.

### Correlation between the effects of stress on transcription of virulence-associated genes and on uptake by amoeba

Previous studies have shown that *ciaB*, *htrA*, and *dnaJ* play important roles in the invasion of *C. jejuni*[11,34,35,38,39,55], but most of these studies involve epithelial cells which have little to no phagocytic abilities. The effect of *ciaB*, *htrA* and *dnaJ* on interaction with amoeba in which entry is based on phagocytosis remained to be established. Our working hypothesis was that transcriptional effects triggered on virulence-associated genes by pre-exposure to stress may affect subsequent interactions with amoeba, even if they did not affect bacterial viability. Therefore, we examined whether down- or up- regulation of virulence-related genes correlated with decreased or increased bacterial uptake and/or intra-amoeba survival, with the understanding that correlation does not imply direct causality.

Overall, our data only showed good correlation between down-regulation of transcription of the three genes investigated (although overall small) and reduced uptake by amoeba for the starvation stress. One may argue that these data reflect the fact that starved bacteria do not have the resources necessary to alter their protein expression patterns in response to further stress (amoeba killing machinery) so that the kinetics of killing are altered. A resulting faster intracellular killing occurring during the 1 h-long gentamicin treatment could explain the apparent lower uptake values. However, ~20% of starved bacteria recovered at T_0_ after gentamicin treatment were recovered at 5 h. This is greater than observed for the heat-stressed bacteria for which the 5 h recovery was only 10% of the bacteria recovered at T_0_, and for which no effect on uptake was detected at T_0_. Therefore, the lower recoveries observed after nutrient stress immediately after gentamicin treatment indicate decreased uptake and not enhanced initial killing.

For the three other stresses tested, we did not observe any clear correlation between gene transcription and uptake by amoeba. This could indicate that the genes may be more important for intracellular survival than for uptake, which we demonstrated with the *htrA* mutant.

### Effect of pre-exposure to stress on intracellular survival in amoeba

The novelty of this study is that we investigated if pre-exposure to stressful conditions may prime the bacteria for resistance to further intracellular stress. The bacteria that had been pre-exposed to low nutrient, heat and osmotic stress were more sensitive to intracellular killing than control *C. jejuni* as seen at 5 h post gentamicin treatment. These results indicate that exposure of *C. jejuni* to these stresses prior to interactions with amoebae not only did not prime the bacteria to fight off the amoebae killing machinery, but also strongly compromised their ability to survive within the amoebae. These findings are consistent with previous data showing that pre-exposure of *C. jejuni* to environmental stresses (except oxidative stress) did not promote its survival within Caco-2 cells or macrophages [45,47]. Heat-stressed bacteria were taken up at non-stressed levels but did not survive any better than starved or osmotically-stressed bacteria that had decreased uptake. This suggests that uptake and intracellular survival rely on distinct properties of the bacteria and that the impact of each stress on either step (uptake or survival) is likely dependent on the repertoire of genes targeted by the transcriptional regulation response elicited by each stress.

## Conclusions

The data presented indicate that environmental stresses such as nutrient starvation, heat exposure and hyper-osmolarity reduced the survival of *C. jejuni* in the absence of amoeba and also reduced its intra-amoeba survival. Starvation and, to a lower extent, osmotic stress also reduced bacterial uptake by amoebae. The observed changes were not correlated directly with stress-induced changes in transcription of virulence-associated genes of *C. jejuni* except for the starvation stress. Oxidative stress had no impact on bacterial survival in the absence of amoeba or on any aspects of amoeba/bacteria interactions, suggesting that *C. jejuni* is well equipped to fight off a moderate oxidative stress and that this pre-exposure does not enhance its ability to respond to further intracellular oxidative damage. Overall, pre-exposure to stress in the outside environment does not seem to prime the bacteria for resistance against further insult by the amoeba killing machinery.

## Methods

### Microorganisms and culture conditions

The reference strain *C. jejuni* NCTC 11168 (ATCC 700819) used in this study was obtained from the American Type Culture Collection. The *htrA* mutant was a kind gift from Prof. Hanne Ingmer (University of Copenhagen, Denmark) and was previously described [39]. Amoeba reference strain *A. castellanii* ATCC 30234 was obtained from the American Type Culture Collection. All bacterial and amoeba culture conditions were as described previously [27].

### Stress conditions

*C. jejuni* cells were grown in microaerobic conditions at 37°C on blood agar plates overnight to the log phase, collected by centrifugation at 3,300 g for 10 min, and washed twice in Phosphate buffered saline (PBS). The bacterial pellet was resuspended in Brucella broth and adjusted to an OD_600_ of 1. This corresponded to ~ 4.5 × 10^8^ CFU/ml. Oxidative and heat stress assays were performed as previously described with slight modifications [13]. Briefly, for oxidative stress assays, bacterial cells were exposed to 10 mM hydrogen peroxide for 15 min. For heat stress assays, bacterial cells were resuspended in 3 ml Brucella broth and incubated at 42°C for 30 min and shifted to 55°C for 3 min. For the osmotic stress assay, *C. jejuni* cells were resuspended in 3 ml Brucella broth supplemented with 1.5% NaCl and incubated at 37°C in microaerobic conditions for 5 h. For low nutrient stress assays, *C. jejuni* cells were grown in microaerobic conditions at 37°C on blood agar plates overnight, collected by centrifugation at 3,300 g for 10 min, and washed twice with amoeba buffer. Amoeba buffer was 4 mM MgSO_4_.7H_2_O, 0.4 mM CaCl_2_, 0.05 mM Fe(NH_4_)_2_(SO_4_)_2_.6H_2_O, 2.5 mM Na_2_HPO_4_.7H_2_O, 2.5 mM KH_2_PO_4_, 0.1% sodium citrate dihydrate, pH 6.5 [60]. The bacteria were resuspended in 3 ml amoeba buffer and incubated at 37°C in microaerobic conditions for 5 h as described before [6]. A non-stressed *C. jejuni* culture, that underwent the same preparation steps as treated campylobacters, served as the control. Non-stressed controls were included in all assays. After exposure to each environmental stress, 10-fold serial dilutions of the samples were spotted on blood agar plates (in triplicates) and incubated at 37°C in microaerobic conditions for 36 h until bacterial colonies formed. Three independent experiments were performed, each including control and stress treatment groups.

### RNA extraction and reverse transcription assays

After exposure to each artificial stress, samples were immediately collected for RNA extraction. Total RNA extraction was performed using cetyltrimethylammonium bromide with phenol, chloroform and isoamyl alcohol as previously described [61]. The RNA was then purified using the RNeasy Mini RNA isolation kit (Qiagen, Copenhagen, Denmark) following the manufacturer’s protocol. The RNA was eluted in RNase-free water and was treated with 0.3 U/ml of DNase I Amplification Grade (Invitrogen, Naerum, Denmark) according to the manufacturer’s instruction. The treated RNA was further tested for DNA contamination by qPCR using primers for *ciaB*, *dnaJ*, *htrA* and 16S rRNA (Table 1). The treated RNA was quantified using a NanoDrop 1000 spectrophotometer Thermo Scientific (Saveen Werner ApS, Jyllinge, Denmark). The DNA-free RNA products were transcribed to complementary DNA (cDNA) using the iScript™ cDNA Synthesis Kit (Bio-Rad, CA, USA) with pre-mixed RNase inhibitor and random hexamer primers, according to the manufacturer’s instruction.

**Table 1:** Primers used in this study

**Primer names**	**Primer sequences (5**^**′**^**-3**^**′**^**)**	**Amplicons (bp)**	**References**
16S RNA*-F*	AACCTTACCTGGGCTTGATA		
16S RNA*-R*	CTTAACCCAACATCTCACGA	122	[34]
*ciaB-F*	ATATTTGCTAGCAGCGAAGAG		
*ciaB-R*	GATGTCCCACTTGTAAAGGTG	157	[34]
*dnaJ-F*	AGTGTCGAGCTTAATATCCC		
*dna-R*	GGCGATGATCTTAACATACA	117	[34]
*htrA-F*	CCATTGCGATATACCCAAACTT		
*htrA-R*	CTGGTTTCCAAGAGGGTGAT	130	This study

### Primer design and quantitative real-time PCR (qPCR) conditions

The sequences of all primers used in this study are listed in Table 1. The *ciaB, dnaJ* and 16S rRNA primers were obtained from a previous study [34] and the *htrA* primers were designed and validated in this study following the same parameters and procedures as for all others.

qPCR assays were carried out in an Mx3005P thermocycler (Strategene, Hørsholm, Denmark). The PCR mixtures (25 μl) contained 5 μl cDNA, 12.5 μl of 2× PCR master mix (Promega, Nacka, Sweden), 400 nM of each primer and 50000× diluted SYBR green (Invitrogen). The qPCR conditions were as recommended by the SYBR green manufacturer and consisted of an initial denaturation at 94°C for 5 min; followed by 45 cycles of denaturation at 94°C for 15 s, annealing at 52°C for 20 s, and extension at 72°C for 15 s; followed by an elongation step at 72°C for 3 min. In every qPCR analysis, a negative control (5 μl of water) and a positive DNA control (5 μl) of *C. jejuni* DNA (2 ng/μl) were included. Each specific PCR amplicon was verified by the presence of both a single melting-temperature peak and a single band of expected size on a 2% agarose gel after electrophoresis. CT values were determined with the Mx3005P software (Strategene). The relative changes (x-fold) in gene expression between the induced and calibrator samples were calculated using the 2^−ΔΔCT^ method as previously described [62]. The 16S rRNA gene was used as the reference gene as previously described [34,49]. qPCR assays were performed using cDNA without dilution from three different RNA extracts of three independent experiments.

### Amoeba infection assays and determination of survival of intracellular bacteria

Co-cultures of *C. jejuni* with monolayers of amoeba cells were performed in 6-well tissue plates (BD, Mississauga, ON, Canada) seeded at a density of 2 × 10^6^ amoeba cells per well and with a multiplicity of infection (MOI) of ~100 bacterial cells per amoeba as described in detail previously [27]. This corresponds to inoculation with ~ 2 × 10^8^ bacteria per well. Except for the controls, the bacteria used had been pre-treated with the stresses described above, before inoculation into the wells. The media for infection assays was amoeba buffer (see composition above). The co-culture was incubated for 3 h at 25°C in aerobic conditions. This temperature is the optimal temperature for amoebae and mimics the environmental conditions found in broiler houses and natural environments [26]. Intracellular survival was assessed using the gentamicin protection assay that we optimized previously [27]. The infected amoeba monolayers were then lyzed with Triton X-100 at 0, 5 and 24 h after gentamicin treatment and the lysate was serially diluted for spot plating to determine the number of intracellular bacteria by bacterial colony forming unit counting. All experiments were carried out in triplicate (3 independent experiments with triplicates in each, and all data obtained were averaged to generate the figures). The number of surviving bacteria was expressed as the % of the inoculum used for co-culture with amoeba, based on bacterial viability data obtained after exposure to each stress.

### Confocal laser scanning microscopy (CLSM) and Transmission electron microscopy (TEM)

Conditions used in this study for CLSM and TEM were described in detail previously [27]. In summary, for CLSM, the bacteria were stained with CelltrackerTM Red CMTPX (Invitrogen, Burlington, ON, Canada) before interactions with amoeba (but after stress exposure), and acidic vacuoles of infected *A. castellanii* monolayers were stained with LysoSensorTM Green DND-189 (Invitrogen, Burlington, ON, Canada). Live cell imaging was performed using a × 63 oil lens with a numeric aperture of 1.2. LysoSensor Green DND-189 was excited at 488 nm with an Argon laser and CellTracker Red CMTPX was excited at 543 nm with a helium-neon laser. Spectral bleed through was tested and prevented using the sequential line scan function. Images of 512 × 512 pixels were taken at a frame rate of 0.5 fps. The pinhole was set at the smallest to get a maximum level of confocality. Confocal microscopy was done at the gap junction facility of the University of Western Ontario, Canada.

For TEM, the infected amoebae were fixed with glutaraldehyde in sodium cacodylate buffer and post-fixed in osmium tetroxide as described previously [27]. After dehydration, the samples were embedded in Epon. Ultrathin sections were collected on one-hole copper grids, and stained with uranyl acetate and lead citrate. Sections were examined with a Philips CM 100 TEM (Eindhoven, Holland) and images were recorded with an OSIS Veleta 2 k × 2 k CCD camera at the Core Facility for Integrated Microscopy of the University of Copenhagen, Denmark.

### Statistical analysis

A Student’s *t*-test (run with Excel software) was used to compare the experimental groups that were subjected to various stresses and the non-stressed controls. P-values of <0.05 were considered statistically significant.

## Competing interests

The authors declare that they have no competing interests.

## Author’s contributions

XTB performed all experiments, prepared all the figures, and wrote a preliminary draft of the manuscript. CC supervised part of the experiments and advised on all data interpretation. She performed extensive editing of the manuscript and rewrote several sections. KQ and XTB performed TEM experiments. AW and DDB advised for and supervised directly part of the study and edited a late version of the manuscript. They also provided funding for most of the study. All authors read and approved the final manuscript.
